# Actively Targeting Redox-Responsive Multifunctional Micelles for Synergistic Chemotherapy of Cancer

**DOI:** 10.1021/acsomega.3c09817

**Published:** 2024-07-29

**Authors:** Haile Fentahun Darge, Kefyalew Dagnew Addisu, Hsieh-Chih Tsai, Yihenew Simegniew Birhan, Endris Yibru Hanurry, Tefera Worku Mekonnen, Hailemichael Tegenu Gebrie, Vinothini Arunagiri, Darieo Thankachan, Tsung-Yun Wu, Juin-Yih Lai, Hao-Ming Chang, Chun-Chiang Huang, Szu-Yuan Wu

**Affiliations:** † Graduate Institute of Applied Science and Technology, National Taiwan University of Science and Technology, Taipei 10607, Taiwan; ‡ College of Medicine and Health Science, Bahir Dar University, P.O. Box 79, Bahir Dar 00000, Ethiopia; § Centre for Ocular Research & Education (CORE), School of Optometry and Vision Science, University of Waterloo, 200 Columbia St W., Waterloo N2L 3W8, Canada; ∥ Institute of Technology, Bahir Dar University, P.O. Box 79, Bahir Dar 00000, Ethiopia; ⊥ Advanced Membrane Materials Center, National Taiwan University of Science and Technology, Taipei 10607, Taiwan; # R&D Center for Membrane Technology, Chung Yuan University, Chung-Li 320, Taiwan; ∇ Department of Chemistry, College of Natural and Computational Sciences, Debre Markos University, P.O. Box 269, Debre Markos 00000, Ethiopia; ○ School of Medicine, Health Science College, Addis Ababa University, P.O. Box 1176, Addis Ababa 00000, Ethiopia; ◆ Division of General Surgery, Tri-Service General Hospital, National Defense Medical Center, Taipei 114, Taiwan; ¶ Taiwan Instrument Research Institute, National Applied Research Laboratories, Hsinchu 300, Taiwan; ◮ Department of Food Nutrition and Health Biotechnology, College of Medical and Health Science, Asia University, Taichung 413, Taiwan; ◭ Big Data Center, Lo-Hsu Medical Foundation, Lotung Poh-Ai Hospital, Yilan 256, Taiwan; ⧩ Division of Radiation Oncology, Department of Medicine, Lo-Hsu Medical Foundation, Lotung Poh-Ai Hospital, Yilan 256, Taiwan; ⧨ Department of Healthcare Administration, College of Medical and Health Science, Asia University, Taichung 413, Taiwan; ◁ Cancer Center, Lo-Hsu Medical Foundation, Lotung Poh-Ai Hospital, Yilan 256, Taiwan; ◀ Graduate Institute of Business Administration, Fu Jen Catholic University, Taipei 242, Taiwan; ▷ Centers for Regional Anesthesia and Pain Medicine, Taipei Municipal Wan Fang Hospital, Taipei Medical University, Taipei 110, Taiwan

## Abstract

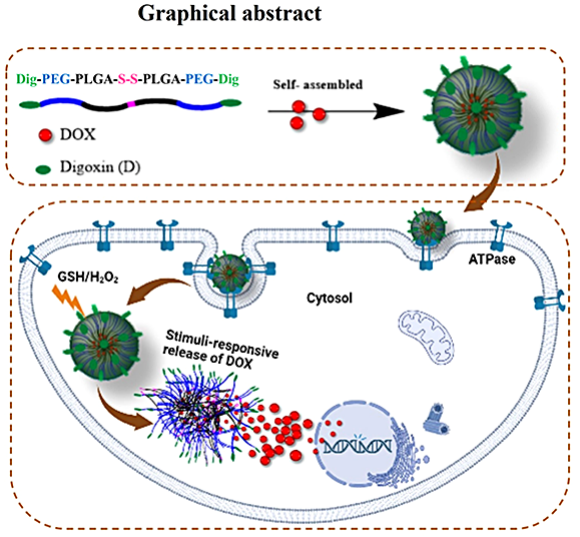

Stimuli-responsive polymeric micelles decorated with cancer biomarkers represent an optimal choice for drug delivery applications due to their ability to enhance therapeutic efficacy while mitigating adverse side effects. Accordingly, we synthesized a digoxin-modified novel multifunctional redox-responsive disulfide-linked poly(ethylene glycol-*b*-poly(lactic-*co*-glycolic acid) copolymer (Bi(Dig–PEG-PLGA)-S_2_) for the targeted and controlled release of doxorubicin (DOX) in cancer cells. Within the micellar aggregate, the disulfide bond confers redox responsiveness, while the presence of the digoxin moiety acts as a targeting agent and chemosensitizer for DOX. Upon self-assembly in aqueous solution, Bi(Dig–PEG-PLGA)-S_2_ formed uniformly distributed spherical micelles with a hydrodynamic diameter (*D*_*h*_) of 58.36 ± 0.78 nm and a zeta potential of −24.71 ± 1.01 mV. The micelles exhibited desirable serum and colloidal stability with a substantial drug loading capacity (DLC) of 6.26% and an encapsulation efficiency (EE) of 83.23%. In addition, the release of DOX demonstrated the redox-responsive behavior of the micelles, with approximately 89.41 ± 6.09 and 79.64 ± 6.68% of DOX diffusing from DOX@Bi(Dig–PEG-PLGA)-S_2_ in the presence of 10 mM GSH and 0.1 mM H_2_O_2_, respectively, over 96 h. Therefore, in HeLa cell lines, DOX@Bi(Dig–PEG-PLGA)-S_2_ showed enhanced intracellular accumulation and subsequent apoptotic effects, attributed to the targeting ability and chemosensitization potential of digoxin. Hence, these findings underscore the promising characteristics of Bi(Dig–PEG-PLGA)-S_2_ as a multifunctional drug delivery vehicle for cancer treatment.

## 1 Introduction

Cancer is one of the most challenging and complex diseases to treat and persists as a major global health concern. The heterogeneity of cancer inter- and intratumoral pathophysiology has made it more challenging to achieve a desirable therapeutic outcome through conventional therapeutic approaches.^1−3^ Chemotherapy is often prescribed as a supportive treatment for various types of malignancies as it efficiently inhibits their proliferation and impedes metastasis. However, the nonspecific distribution of such chemotherapeutic drugs throughout the body leads to serious side effects on healthy cells.^4,5^ Thus, there has been a growing focus on improving the pharmacokinetic attributes of chemotherapeutic agents, such as enhancing solubility and selectivity while maintaining their chemotherapeutic potential, in recent years.^6−8^ In this regard, drug delivery systems (DDS) utilizing nanoparticles, micelles, gels, liposomes, polymeric prodrugs, and other polymeric functional materials have been developed. These systems offer advantages, such as biocompatibility and versatility in surface modification and facilitate the selective accumulation and controlled release of chemotherapeutic agents in the vicinity of cancer cells.^9,10^ Moreover, they have the potential to prevent sequestering payloads by the phagocytic system, reduce accumulation in organs like the liver and spleen, and prolong systemic circulation, thereby enhancing anticancer effects.^11^

Polymeric micelles, often made of amphiphilic block copolymers, are the most preferred nanocarriers for delivering hydrophobic chemotherapeutic agents, owing to their hydrophobic core, capable of solubilizing and encapsulating water-insoluble agents.^12^ Moreover, the hydrophilic shell serves as a hydration barrier, increasing colloidal stability and prolonging the systemic circulation by avoiding rapid renal excretion and uptake by the reticuloendothelial system (RES). Subsequently, this prolongs the accumulation of the micellar assemblies in tumor tissue through the enhanced permeability retention (EPR) effect.^13^ Achieving therapeutic drug concentration in tumor tissue is fundamental to counteract the effects of membrane transporters like *P*-glycoprotein, which are implicated in the efflux of drugs and suboptimal intracellular concentration, resulting in the emergence of multidrug resistance toward the payloads.^14,15^ Surface functionalization of polymeric micells via the physical or chemical conjugation of different cancer cell biomarkers, such as antibodies, transferrin, folic acid, aptamers, RGD peptides, etc., can enhance the selective cellular uptake of drug-loaded micelles through receptor-mediated endocytosis.^10,16,17^ Furthermore, the spatiotemporal release of payloads in the intracellular compartment of cancer cells is equally important to elicit a prompt cancer-killing effect. Most micellar aggregates suffer from premature payload release in the systemic circulation before reaching the target tissue, partly due to diffusion and/or biodegradation-triggered release kinetics.^18^ This premature release leads to undesirable side effects, suboptimal therapeutic outcomes, and cancer recurrence.^19^ Thus, the incorporation of stimuli-responsive moieties that respond to external or internal stimuli (such as *p*H, temperature, reduction, oxidation, light, enzyme, *etc*.) in micellar systems has garnered significant attention in recent decades. These stimuli-responsive micellar aggregates remain intact in the bloodstream and selectively release their cargo at pathological sites when exposed to stimuli specific to tumor tissue.^20−23^ Overall, the fabrication of tumor targeting and stimuli-responsive micellar systems holds promise to address the prospect of premature payload release and nonselective biodistribution of anticancer drugs in the body.

Taking this into account, we synthesized a novel digoxin-modified redox-responsive Bi(Digoxin–PEG-PLGA)-S_2_ block copolymer, hereafter referred to as Bi(Dig–PEG-PLGA)-S_2_. This copolymer self-assembled into micelles with a PLGA–S–S-PLGA core and digoxin-PEG shell, intended for the targeted delivery of doxorubicin (DOX) to cancer cells. The presence of digoxin as a targeting moiety facilitates selective and site-specific internalization of DOX-loaded micelles, as digoxin often interacts with Na^+^/K^+^ ATPase, which is overexpressed on the plasma membrane of cancer cells. Moreover, the use of digoxin has synergistic therapeutic effects owing to its sensitization of cancer cells toward chemodrugs.^24,25^ Furthermore, a redox-responsive disulfide moiety was incorporated to achieve spatiotemporal release of DOX within cancer cells. The disulfide bond exhibits profound sensitivity due to its relatively lower bond energy (240 kJ/mol)^26,27^ and the appealing difference in the concentration of glutathione (GSH), a reducing agent, and hydrogen peroxide (H_2_O_2_), an oxidizing agent, between cancer (2–10 mM GSH and 0.1 mM H_2_O_2_) and normal cells (2–10 μM GSH and 20 nM H_2_O_2_).^27−29^ Following receptor-mediated endocytosis and intracellular accumulation in cancer cells, the DOX-loaded Bi(Dig–PEG-PLGA)-S_2_ micelles (DOX@Bi(Dig–PEG-PLGA)-S_2_) can swell or disassemble, subsequently releasing DOX due to the oxidation and reduction of the S–S bond, which trigger phase change in the micellar core.^26^ In this study, fluorescence microscopy, MTT assay, and flow cytometry analysis were used to examine the *in vitro* cellular uptake and cytotoxic effect of DOX-loaded Bi(HOOC–PEG-PLGA)-S_2_ (DOX@Bi(HOOC–PEG-PLGA)-S_2_) and DOX@Bi(Dig–PEG-PLGA)-S_2_ micelles against HeLa cells. The results revealed that DOX@Bi(Dig–PEG-PLGA)-S_2_ exhibited superior cellular internalization and cancer cell inhibition compared to DOX@Bi(HOOC–PEG-PLGA)-S_2_. Moreover, the effect of DOX@Bi(Dig–PEG-PLGA)-S_2_ on apoptosis and necrosis of HeLa cells was comparable to that of cells treated with free DOX.

## 2 Material and Methods

### 2.1 Materials

Polyethylene glycol (COOH-PEG–OH, *Mn* = 2 kDa), glycolide (99%), D, l-lactide (99%), stannous octoate (Sn(Oct)_2_, 95%), digoxin, *N,N*′-dicyclohexylcarbodiimide (DCC), 4-dimethylaminopyridine (DMAP), anhydrous tetrahydrofuran (THF, ≥ 99.9%), dichloromethane (DCM, ≥ 99.8%), diethyl ether (≥99.7%), triethylamine (TEA), paraformaldehyde (95%), glutathione (GSH), hydrogen peroxide (H_2_O_2_), and dimethyl sulfoxide (DMSO, ≥ 99.9%) were purchased from Sigma-Aldrich and used without further purification step. Dulbecco’s modified Eagle’s medium (DMEM), fetal bovine serum (FBS), penicillin, and trypsin-EDTA (0.25%), 3-(4,5-dimethylthiazol-2yl)-2,5-diphenyl-tetrazolium bromide (MTT, 98%), and sterilized phosphate-buffered saline (PBS) were purchased from Gibco (Carlsbad, CA), while doxorubicin hydrochloride (DOX·HCl) was purchased from Cayman Chemical Co., Ltd. Alexa fluor 488 annexin V and propidium iodide (PI) were purchased from Thermo Fisher Scientific. HeLa cells (human cervical carcinoma cells) and NIH-3T3 cells (mouse embryonic fibroblast cells) were obtained from the BioResource Collection and Research Center (Hsinchu, Taiwan).

### 2.2 Synthesis of HOOC–PEG-PLGA

The block copolymer HOOC–PEG-PLGA was synthesized by ring-opening copolymerization (ROP) of GA and LA using HOOC-PEG–OH as an initiator and Sn(Oct)_2_ as a catalyst (Scheme S1a) following ref. (30) with a slight modification. Briefly, 1.25 mmol of HOOC-PEG–OH was placed into a two-neck reaction flask and dried in a vacuum oven at 110 °C for 3 h to eliminate traces of water. The temperature was reduced to 90 °C, and 32.25 mmol of LA and 10.75 mmol of GA (LA:GA ratio = 3) were added under nitrogen protection and maintained for 30 min to remove the residual moisture. Then, 150 μL of Sn (Oct)_2_ catalyst was injected into the reaction mixture and continuously stirred at 150 °C for 24 h. Finally, the crude product was purified by dissolving it in a small amount of DCM and precipitating in large excess cold diethyl ether (repeated three times). The residual solvent was completely eliminated from the precipitate by oven drying under a vacuum at 45 °C for 48 h. The target product was stored in a refrigerator at below 10 °C.

### 2.3 Synthesis of Bi(HOOC–PEG-PLGA)-S_2_

A disulfide-linked HOOC–PEG-PLGA copolymer (Bi(HOOC–PEG-PLGA)-S_2_) was prepared by coupling reaction in the presence of DMAP and DCC (Scheme S1b).^26,30^ Briefly, 0.08 mmol of HOOC–PEG-PLGA copolymer and 0.23 mmol of DMAP were dissolved in a anhydrous THF (20 mL) in a two-neck reaction flask. Then, the mixture was stirred at 0 °C under a nitrogen atmosphere until it was completely dissolved. After 30 min, 0.04 mmol of 3,3′-dithiodiproponic acid (disulfide linker) and 0.23 mmol of DCC were added and stirred at 0 °C and room temperature (∼25 °C) for 4 and 72 h, respectively. The resulting product was purified by membrane dialysis with MWCO = 2 kDa against THF for 8 h and by deionized (DI) water for 48 h (exchanged every 6 h).

### 2.4 Synthesis of Bi(Dig–PEG-PLGA)-S_2_ Copolymer

Bi(Dig–PEG-PLGA)-S_2_ was synthesized from Bi(HOOC–PEG-PLGA)-S_2_ and digoxin in the presence of catalytic amounts of DMAP and DCC (Scheme S1c).^26,30^ Shortly, 0.08 mmol of Bi(HOOC–PEG-PLGA)-S_2_ copolymer and 0.23 mmol of DMAP were dissolved with anhydrous THF (20 mL) in a two-neck reaction flask. Then, the mixture was stirred at 0 °C under a nitrogen atmosphere. After 30 min, 0.2 mmol of digoxin and 0.23 mmol of DCC were added and stirred at 0 °C and room temperature (∼25 °C) for 4 and 72 h, respectively. The resulting Bi(digoxin–PEG-PLGA)-S_2_ was purified by membrane dialysis with MWCO = 2 kDa against THF for 24 h and DI water for 48 h (exchanged every 6 h) to remove unbound digoxin, residual DMAP, and DCC.

### 2.5 Characterization

The chemical structures of the synthesized copolymers were verified by ^1^H NMR using a Varian Unity-600 NMR spectrometer (Varian, Inc., CA, USA) at 600 MHz. In addition, the molecular weights of the respective copolymers were determined using the advanced polymer chromatographic (APC) technique using a THF column (ACUITY APC system). For this purpose, 2 mg/mL of HOOC–PEG-PLGA and Bi(HOOC–PEG-PLGA)-S_2_ samples were prepared using THF as a solvent, and the molecular weight and polydispersity index (*Đ*) were measured at 45 °C at a flow rate of 0.8 mL/min using polystyrene as an internal standard for molecular weight calibration. Moreover, the particle size surface charge or zeta (ζ)-potential of the micelles was measured using a Dynamic Light Scattering (DLS) analyzer (Horiba Zeta sizer-100 system, UK) with a scattering angle of 90° at a temperature of 25 °C in triplicate, and the results were expressed as mean ± SD. Field-Emission Scanning Electron Microscopy (FE-SEM, JSM 6500F, JEOL) was also used to assess the morphology and estimate the particle size of the micelles. Herein, a droplet of diluted micelle solution was spin-coated on a silicon substrate and vacuum-dried for 24 h, followed by platinum coating for 10 min before FE-SEM image scanning. The absorbance and emission spectra of the different samples prepared during drug loading, critical micelle concentration (CMC) determination, *etc*., were also determined using UV–vis spectroscopy (JASCO V-730) and photoluminescence spectroscopy (JASCO V-330), respectively.

### 2.6 Determination of Critical Micelle Concentration

The CMC of the (Bi(Dig–PEG-PLGA)-S_2_) copolymer in aqueous solution was determined by the pyrene fluorescent probe method using a fluorescence spectrophotometer (JASCO V-330). Briefly, serial concentrations of 1 × 10°, 1 × 10^–1^, 5 × 10^–2^, 1 × 10^–2^, 5 × 10^–3^, 1 × 10^–3^, and 5 × 10^–4^ mg/mL of Bi(Dig–PEG-PLGA)-S_2_ copolymer solutions were prepared using the dilution method and stored overnight at 10 °C. In the meantime, pyrene was dissolved in acetone to make a stock solution with a concentration of 2 × 10^–4^ M. From this stock solution, 20 μL was transferred into seven different amber glass vials, and the acetone was evaporated in the dark for 12 h. The serial concentrations of the different copolymer solutions were transferred to the respective pyrene-containing vials to obtain a final pyrene concentration of 2 × 10^–5^ M and then continuously sonicated for 30 min. After stabilizing overnight at room temperature, the fluorescence emission spectra of pyrene were scanned between 350 and 440 nm with an excitation wavelength of 336 nm and an excitation and emission slit width of 2.5 nm. Finally, the CMC of the copolymer was computed by plotting the intensity ratio of the first peak at 372 nm and the third peak at 383 nm (I372/I383) of the pyrene emission spectra against the copolymer concentrations (log^C^). The intersection point of the vertical and horizontal tangent lines of the curve through the points of low concentration is considered as the CMC of the copolymer.^26,31^

### 2.7 Preparation of Blank and DOX-Loaded Micelles

The self-assembly behavior of Bi(HOOC–PEG-PLGA)-S_2_ and Bi(Dig–PEG-PLGA)-S_2_ into micellar aggregates was investigated based on the reported protocols with minor modifications.^31,32^ A 5 mg/mL concentration of copolymer in DMSO was prepared, and the probe was sonicated for 5 min by adding water dropwise. Then, it was transferred into 4-fold ultrapure water drop by drop with gentle stirring and proceeded overnight. DMSO was removed by dialysis against DI water for 48 h, during which water was exchanged at 4 h intervals. Finally, the size and surface charge of the micellar solution were estimated via DLS. Similarly, DOX-loaded micelles were prepared by the solvent exchange method with dialysis.^30,33^ DOX·HCl (4 mg) was dissolved in 2 mL of DMSO and neutralized with 5 μLTEA to obtain a free and hydrophobic DOX solution. It was subsequently mixed with copolymers (40 mg dissolved in 2 mL of DMSO), probe-sonicated for 5 min, and added dropwise to 15 mL of PBS under vigorous stirring for 36 h. The DMSO and unbound DOX were then removed by membrane dialysis (MWCO = 1 kDa) against DI water for 24 h (water exchanged at every 4 h) (Figure S3). Finally, the DOX-loaded micellar solutions were collected, and the absorbance of DOX was determined at 485 nm via UV–vis spectrophotometer to compute the drug-loading capacity (DLC) and encapsulation efficiency (EE) using a pre-established DOX calibration curve (Figure S4) using the following formula
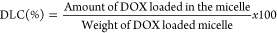

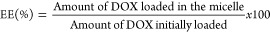


### 2.8 Colloidal and Serum Stability of Micelles

The colloidal stability of Bi(Dig–PEG-PLGA)-S_2_ micelles was investigated using the dilution method. It is based on the dilution of the micellar solution against a large volume of solvent, usually PBS, which represents the volume of the entire systemic circulation.^31^ Thus, a 5 mg/mL micellar solution was prepared, and the volume was adjusted to 1000 folds by diluting with PBS. Then, the change in hydrodynamic diameter (*D*_*h*_) as a function of time was measured in triplicate every 24 h for 7 days. In addition, the serum stability of Bi(Dig–PEG-PLGA)-S_2_ micelles was assessed by incubating freshly prepared micelles in 50% FBS (used to simulate biological fluid) and monitoring the change in *D*_*h*_ of the micelles as a function of time.

### 2.9 Redox-Responsive Drug Releasing Behavior of Micelles

The *in vitro* drug-releasing kinetics of DOX@Bi(Dig–PEG-PLGA)-S_2_ micelles were investigated in a simulated cancer redox environment and at normal physiological conditions (PBS and *p*H 7.4). In brief, a fixed weight of DOX@Bi(Dig–PEG-PLGA)-S_2_ micelles in 2 mL of PBS was placed in a dialysis bag (MWCO = 1 kDa) and immersed into 10 mL of release medium (PBS of pH 7.4, 5 mM GSH, 10 mM GSH, and 0.1 mM H_2_O_2_) in a 20 mL vial. Then, it was placed in an orbital shaker incubator (Yihder Orbital Shaker, LM-420D) by maintaining the temperature at 37 °C with mild agitating at 100 rpm. At a predetermined time, 1, 2, 3, 4, 6, 12, 24, 48, 72, and 96 h, 3 mL of each release medium was collected from the vials for UV–vis measurement of DOX and replenished with an equal volume of new medium. Finally, the amount of DOX released from the micelles was calculated using the pre-established standard calibration curves of free DOX.

### 2.10 Cellular Uptake of DOX-Loaded Micelles

The cellular trafficking of DOX@Bi(HOOC–PEG-PLGA)-S_2_ and DOX@Bi(Dig–PEG-PLGA)-S_2_ micelles against HeLa cell lines was monitored using a fluorescence scanning microscope (iRiSTM Digital Cell Imaging System from Logos Biosystem).^30^ The HeLa cells were seeded in confocal dishes (2 × 10^5^ cells/dish) with complete DMEM medium (containing 10% (v/v) FBS and 1% (v/v) streptomycin) and incubated for 24 h at 37 °C and 5% CO_2_ in a humidified incubator. The cells were then treated with free DOX, DOX@Bi(HOOC–PEG-PLGA)-S_2_, and DOX@Bi(Dig–PEG-PLGA)-S_2_ at equivalent DOX concentrations of 3 μg/mL. At predetermined time points (4 and 12 h), the culture medium was removed, washed three times with PBS, and stained with DAPI (300 nM) for 30 min to stain the nuclei of HeLa cells. Then, fluorescence cellular images were taken to trace the internalization of DOX@Bi(HOOC–PEG-PLGA)-S_2_ and DOX@Bi(Dig–PEG-PLGA)-S_2_ into the cells.

### 2.11 *In Vitro* Cytotoxicity of the Blank and DOX-Loaded Micelles

The biocompatibility of Bi(Dig–PEG-PLGA)-S_2_ was studied in normal cell lines (NIH-3T3 cells and embryonic fibroblast cells of the NIH/Swiss mouse) and cancer cell lines (HeLa cells and human cervical carcinoma cells). The respective cell lines were seeded in 96-well plate at a density of 1 × 10^4^ cells/wall in triplicate using complete culture medium (DMEM supplemented with 10% FBS and 1% streptomycin) and incubated for 24 h at 37 °C in a humidified atmosphere with 5% CO_2_. Then, the old medium was replenished with fresh medium containing Bi(Dig–PEG-PLGA)-S_2_ at concentrations of 10, 20, 40, 60, 80, 100, and 200 μg/mL and incubated for another 24 h. The cells were washed three times with PBS and treated with MTT solution (1 mg/mL medium in each well). The MTT solution was removed after 4 h of incubation, and 100 μL of DMSO was added to each well to dissolve the formazan crystals, and the absorbance was measured at 570 nm using a microplate reader (ThermoMultiskan FC microplate photometer, USA). The viability of NIH-3T3 and HeLa cells was calculated according to the following formula
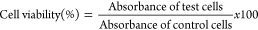


Similarly, the cell viability of DOX@Bi(HOOC–PEG-PLGA)-S_2_ and DOX@Bi(Dig–PEG-PLGA)-S_2_ micelles was determined by the same procedure against NIH-3T3 and HeLa cell lines at an equivalent DOX concentration of 0.5–12.5 μg/mL.

### 2.12 Apoptosis Assays of DOX-Loaded Micelles

Apoptosis and subsequent cell death-inducing capacity of DOX@Bi(HOOC–PEG-PLGA)-S_2_ and DOX@Bi(Dig–PEG-PLGA)-S_2_ micelles werestudied using Alexa fluor 488 annexin-V/PI dual staining assay in accordance with ref. (34,35). Briefly, 2 × 10^5^ HeLa cells were seeded in confocal dishes and incubated for 24 h. After that, the cells were treated with free DOX, DOX@Bi(HOOC–PEG-PLGA)-S_2_, and DOX@Bi(Dig–PEG-PLGA)-S_2_ micelles containing an equivalent DOX concentration of 5.5 μg/mL each for 12 h. The media containing DOX, DOX@Bi(HOOC–PEG-PLGA)-S_2_, and DOX@Bi(Dig–PEG-PLGA)-S_2_ was removed, rinsed with PBS, and incubated for 30 min at room temperature (in the dark), following the addition of Alexa fluor 488 annexin-V (2 μM) and PI (2 μM). Finally, the probes were rinsed with annexin V binding buffer (1X) repeatedly, and fluorescence images were taken using a fluorescent microscope to analyze the apoptotic and dead cells.

### 2.13 Flow Cytometry Analysis of DOX-Loaded Micelles

A fluorescent activated cell sorting (FACS) assay was conducted to quantify the number of live, apoptotic, and necrotic cells. HeLa cells were seeded in 6-well plates (1 × 10^6^ cells/well) and incubated for 24 h at 37 °C and 5% CO_2_. After rinsing with PBS, the cells were treated with PBS (control group), free DOX, DOX@Bi(HOOC–PEG-PLGA)-S_2_, and DOX@Bi(Dig–PEG-PLGA)-S_2_ (5.5 μg/mL) for 12 h. Then, the cells were rinsed with PBS, detached using trypsin, centrifuged (for 5 min at 1200 rpm), and resuspended in 100 μL of annexin V binding buffer (1X). Next, 2 μM of Alexa fluor 488 annexin-V and PI were added and incubated for 20 min at room temperature (in the dark). Finally, the cell suspensions were diluted with annexin-V binding buffer (400 μL), and the live, apoptotic, and necrotic cells were quantified by flow cytometry instrument (FACScan; Becton Dickinson, Heidelberg, Germany).

### 2.14 Statistical Analysis

The data values are reported as the mean ± SD of triplicate experiments. The statistical significance of the variances between the two groups was determined using a two-tailed student *t*-test. A *p*-value <0.05 was considered as statistically significant.

## 3 Results and Discussion

### 3.1 Synthesis and Characterization of Bi(Dig–PEG-PLGA)-S_2_ Copolymer

**Figure 1:**
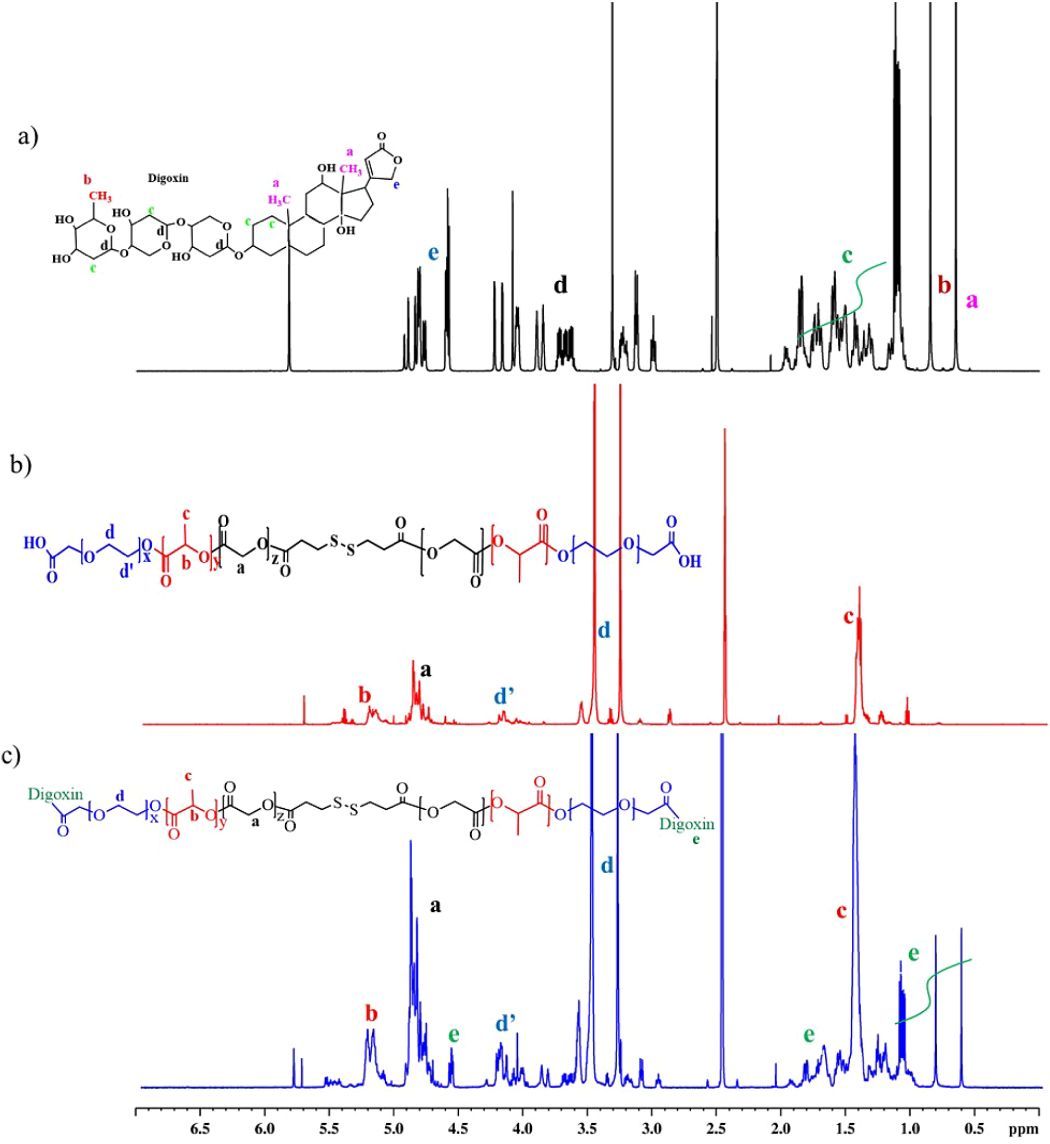
Chemical Shift (ppm) in ^1^H NMR spectroscopy using deuterated dimethyl sulfoxide (DMSO-d6) solvent. a) ^1^H NMR spectra of Bi(Dig–PEG-PLGA)-S_2_, b) Bi(HOOC–PEG-PLGA)-S_2_, and c) Digoxin.

The amphiphilic block copolymer, Bi(Dig–PEG-PLGA)-S_2_, can self-assemble into micellar aggregates with a core–shell structure capable of encapsulating hydrophobic chemotherapeutic agents.^36^ The synthesis of this copolymer involves the following steps: first, ring opening polymerization (ROP) of GA and LA in the presence of HOOC-PEG–OH as an initiator to form HOOC–PEG-PLGA. Next, two equiv of HOOC–PEG-PLGA were linked via 3,3′-dithiodiproponic acid to form Bi(HOOC–PEG-PLGA)-S_2_. Finally, Bi(HOOC–PEG-PLGA)-S_2_ was decorated with digoxin to prepare Bi(Dig–PEG-PLGA)-S_2_ through the DCC/DMAP coupling reaction. During these reactions, DPC and DMAP were used to activate the −COOH group to trigger ester bond formation under dry solvent, anhydrous THF.^26,31^^1^H NMR spectroscopy was used to verify the chemical structures of the synthesized block copolymers. As shown in Figure 1a, the characteristic peak at *δ*_*H*_ 4.9 ppm (a) is ascribed to the CH_2_ protons of GA, while the signals at *δ*_*H*_ 5.3 ppm (b) and 1.5 ppm (c) represent the CH and CH_3_ protons of LA, respectively. Additionally, the peak that appeared between *δ*_*H*_ 3.3–3.6 ppm (d) confirmed the presence of CH_2_ protons of PEG, suggesting the successful synthesis of the HOOC–PEG-PLGA copolymer, which is in agreement with a previous report.^37^ In the case of the the disulfide-linked copolymer, Bi(HOOC–PEG-PLGA)-S_2_, additional peaks *δ*_*H*_ 2.9 and *δ*_*H*_ 3.1 ppm are representative of S–CH_2_-CH_2_-COOH protons of 3,3′-dithiodiproponic acid, verifying the successful conjugation of HOOC–PEG-PLGA and 3,3′-dithiodiproponic acid to form Bi(HOOC–PEG-PLGA)-S_2_ (Figure 1b). When the terminal −COOH moiety of Bi(HOOC–PEG-PLGA)-S_2_ was decorated with digoxin, in addition to the aforementioned proton peaks of HOOC–PEG-PLGA and Bi(HOOC–PEG-PLGA)-S_2_, new peaks that are characteristic of digoxin protons appeared in the ^1^H NMR spectrum, as shown in Figure 1c. The peaks that appeared between *δ*_*H*_ 0.6–1.2 ppm indicated the methyl proton of digoxin. Similarly, the peaks between *δ*_*H*_ 1.5–2.1 ppm and *δ*_*H*_ 6.0 ppm were assigned to the methylene proton of digoxin, signifying the successful chemical conjugation of Bi(HOOC–PEG-PLGA)-S_2_ and the targeting moiety, digoxin. To further confirm the synthesis of block copolymers, an advanced polymer chromatography technique was employed, and an average molecular weight (*Mn*) of 5713 g/mol with a narrow molecular weight distribution (*Đ* = 1.23) was recorded for HOOC–PEG-PLGA (Figure S1b and Table 1). Furthermore, as two equivalents of HOOC–PEG-PLGA copolymers were covalently linked by 3,3′-dithiodiproponic acid to form Bi(HOOC–PEG-PLGA)-S_2_, the molecular weight of the resulting copolymer is expected to double.^26^ Consistently, the average molecular weight of Bi(HOOC–PEG-PLGA)-S_2_ was found to be 10 976 g/mol with unimodal distribution (Table 1). The APC chromatogram reiterated the relatively lower retention time (faster elution) for Bi(HOOC–PEG-PLGA)-S_2_ than the HOOC–PEG-PLGA copolymer, further asserting the efficient synthesis protocols followed and the successful preparation of Bi(HOOC–PEG-PLGA)-S_2_ (Figure S1c).

**Table 1:** Molecular Weight of the Synthesized Copolymer Measured by APC^a^

copolymers	*Mn* (g/mol)	*Mw* (g/mol)	*Mp* (g/mol)	*Đ*
HOOC–PEG-PLGA	5713	7039	5180	1.23
Bi(HOOC–PEG-PLGA)-S_2_	10976	13012	10241	1.18

a *Mn*: number average molecular weight; *Mw*: weight average molecular weight; *Mp*: molecular weight at peak maximum.

### 3.2 Critical Micelle Concentration of Bi(Dig–PEG-PLGA)-S_2_

Above the CMC (a certain concentration), amphiphilic block copolymers undergo self-assembly to form well-defined micelles. This spontaneous entanglement of copolymers in aqueous solution is a reversible thermodynamic process, and strongly depends on the CMC and intermolecular interactions within the micelles. A lower CMC value indicates higher stability of the micellar aggregate, particularly at extreme dilution, making the CMC a crucial measure of polymeric micelle stability.^31,38^ When micelles are administered into the body, their structural integrity may be altered by the large volume of body fluid in the systemic circulation. Micelles with a higher CMC will undergo spontaneous swelling and disassembly to release their payload before reaching the target tissues. Therefore, the CMC determination is of paramount importance in the fabrication of anticancer drug delivery vehicles, specifically micelles. Because of their amphiphilic nature, Bi(HOOC–PEG-PLGA)-S_2_ and Bi(Dig–PEG-PLGA)-S_2_ spontaneously form micelles in an aqueous solution with a hydrophobic PLGA core (that can encapsulate DOX or pyrene) and a hydrophilic PEG shell that interact with the surrounding medium. Encapsulation of DOX within Bi(HOOC–PEG-PLGA)-S_2_ and Bi(Dig–PEG-PLGA)-S_2_ micelles could improve the solubility and minimize systemic toxicity during therapeutic usage.^31^ In this study, the CMC of the Bi(Dig–PEG-PLGA)-S_2_ copolymer was estimated using a fluorescence spectrophotometer, employing pyrene as the hydrophobic core probe. The CMC of the copolymer was found to be 0.014 mg/mL (Figure S2b), indicating that Bi(Dig–PEG-PLGA)-S_2_ copolymer-based micelles might be stable at extreme dilution and could have longer circulation times without premature release of its cargo.^26^ This CMC value is comparable to those reported for similar amphiphilic block copolymer micelles in previous reports: 0.0091 mg/mL^39^ and 0,0139 mg/mL.^26,40^

### 3.3 Particle Size and Surface Charge of the Micelles

**Figure 2:**
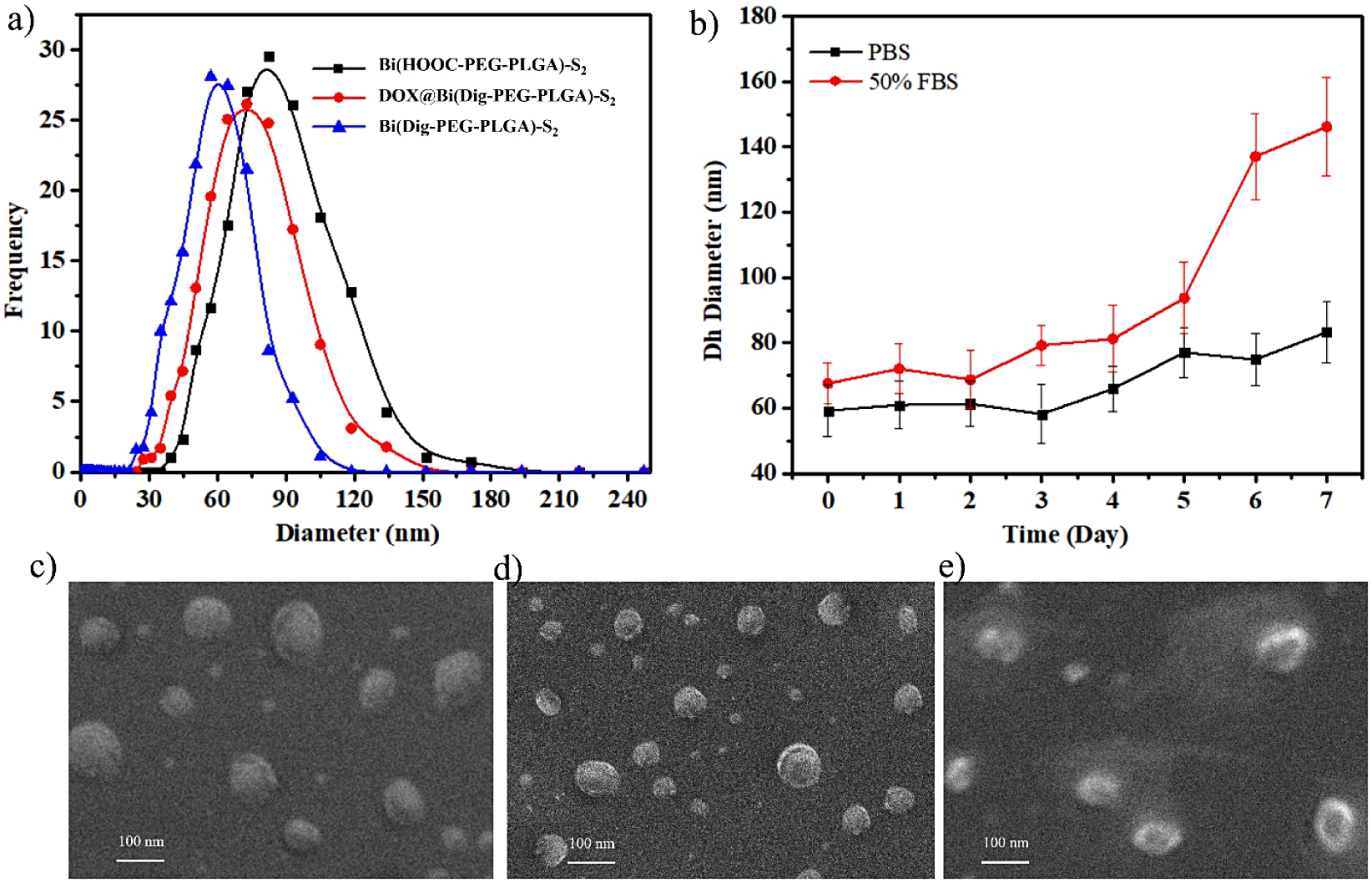
Characterization of the micelles. a) Size of the micelles with DLS, b) stability of Bi(Dig–PEG-PLGA)-S_2_ based micelles in PBS and 50% FBS, c) FE-SEM image of Bi(HOOC–PEG-PLGA)-S_2_, d) Bi(Dig–PEG-PLGA)-S_2_, and e) DOX@Bi(Dig–PEG-PLGA)-S_2_.

The drug delivery potential of polymeric micelles is strongly correlated to their *D*_*h*_ or particle size. Nanosized micelles (≤200 nm) often experienced better extravasation potential through the tight lining of blood vessels and better accumulation in tumor tissue through the EPR effect. Micelles that are too small (≤5 nm) or too large (≥500 nm) may be eliminated from the body through the kidney and RES, respectively. Similarly, the surface charge can also dictate the magnitude of interaction between micelles and cellular structures of cancer cells,^41,42^ thereby affecting the cellular internalization and cancer-killing effect of payloads. The *D*_*h*_ and surface charge of Bi(Dig–PEG-PLGA)-S_2_ micelles were determined using DLS. As shown in Table 2 and Figure 2a, the *D*_*h*_ of the Bi(Dig–PEG-PLGA)-S_2_ micelles was slightly lower than that of the Bi(COOH–PEG-PLGA)-S_2_ micelles, with relatively narrow size distributions (*Đ* ranged from 0.2 to 0.4). The decrease in the size of micelles prepared from Bi(Dig–PEG-PLGA)-S_2_ might be partly attributed to the hydrophobic digoxin terminals that induce tight packing and reduce the size of the micelles.^43^ However, after DOX was loaded in the hydrophobic core of Bi(Dig–PEG-PLGA)-S_2_ micelles, a slight increment in size was noted, indicating that the enclosure of DOX in the micelles’ core increased its volume.^44,45^ Furthermore, the SEM image (Figure 2c**–**e) confirmed the spherical shape of uniformly distributed polymeric micelles. The estimated particle size from SEM images closely matched with the size obtained with DLS measurement, despite the difference in sample preparation. The surface charges (ζ-potential) of both blank and DOX@Bi(Dig–PEG-PLGA)-S_2_ micelles were also analyzed using DLS, and the absolute value of ζ-potential was ranged between 20 and 35 mV (Table 2). This falls within a desirable range for enhancing cellular internalization via endocytosis and preventing self-aggregation during storage.^46−48^ Comparing blank micelle to DOX@Bi(Dig–PEG-PLGA)-S_2_ micelles, a slight shift toward a positive direction in ζ-potential (from −24.71 to −20.67) was observed, which might be due to the presence of an -NH_2_ group in DOX that could ionize into a positive charge in aqueous medium.^49^

**Table 2:** Size and Surface Charge of the Micelles were Determined from DLS Measurements

micelles	*Z*-average (nm)	*Đ*	ζ-potential (mV)
Bi(HOOC–PEG-PLGA)-S_2_	82.12 ± 1.73	0.43 ± 0.22	-35.53 ± 0.62
Bi(Dig–PEG-PLGA)-S_2_	58.36 ± 0.78	0.23 ± 0.06	-24.71 ± 1.01
DOX@Bi(Dig–PEG-PLGA)-S_2_	74.41 ± 1.19	0.31 ± 0.14	-20.67 ± 0.82

### 3.4 Colloidal and Serum Stability of Micelles

The fabrication of stable micelles with prolonged systemic circulation remains a challenging research area in drug delivery science. The efficient accumulation of micelles into the extracellular matrix or cancer niche demands intact and stable aggregates with a longer circulation half-life, ensuring sustained exposure of cells to payloads. However, the structural integrity of polymeric micelles can be compromised by their undesirable interaction with serum proteins and the dilution effect of the systemic circulation. Because the CMC of copolymers can be affected by the large excess volume of the entire plasma fluid in the body,^38,50^ Overall, such detrimental interactions result in disassembly of micelles into polymer chains and rapid clearance of micelles from the circulatory system through the RES.^51,52^ Taking this into account, the colloidal stability of Bi(Dig–PEG-PLGA)-S2 micelles was examined by incubating 5 mg/mL of the micelles in a 1000-fold volume of PBS and 50% FBS, and the change in *Dh* of the micelles was monitored for seven consecutive days. As shown in Figure 2b, micelles incubated with PBS (pH 7.4) showed insignificant changes in *Dh* (60.96 ± 7.22 to 83.27 ± 9.45 nm over 7 days) under physiological conditions (pH 7.4 and 37 °C). Conversely, micelles exposed to 50% FBS remained relatively stable for five consecutive days before experiencing a slight increment in size during the sixth and seventh days. Although the interaction of micelles with the proteins in FBS caused quicker micellar swelling (an increase in *Dh*) compared to that in micelles incubated with PBS alone, the stability period of micelles in 50% FBS was quite promising. This suggests that they may remain intact in the bloodstream and may achieve longer half-life for effective therapeutic outcome.^48^

### 3.5 Drug Encapsulation Potential of Bi(Dig–PEG-PLGA)-S_2_

Polymeric micelles should encamp within a short period of time. Hence, polymeric micelles should possess a relatively higher drug loading and encapsulating efficiency. Bi(HOOC–PEG-PLGA)-S2 and Bi(Dig–PEG-PLGA)-S2 are amphiphilic block copolymers capable of self-assembling into micellar nanostructures in aqueous solution; hence, they can encapsulate hydrophobic drugs, such as DOX, predominantly in the core, and to a lesser extent, at the interface between the hydrophobic and hydrophilic segments. This encapsulation involves a weak hydrophobic interaction between DOX and the core as well as the π–π stacking among DOX molecules.^55^ In this study, DOX was loaded in the hydrophobic core of Bi(HOOC–PEG-PLGA)-S2 and Bi(Dig–PEG-PLGA)-S2 micelles through the dialysis method. The DLC and EE were calculated by measuring the absorbance of DOX-loaded micelles using a UV–vis spectrophotometer and the pre-established calibration curve of DOX (Figure S4). Bi(Dig–PEG-PLGA)-S2 exhibited relatively higher DLC (6.26%) and EE (83.23%) compared to those of Bi(HOOC–PEG-PLGA)-S2 micelles (DLC, 6.01% and EE, 79.19%). This slight variation in DLC between two micellar formulations might be due to the adsorption of DOX molecules on the surface of the micelle due to the favorable π–π interaction with hydrophobic digoxin present in the shell of Bi(Dig–PEG-PLGA)-S2.^53−55^ Overall, the DLC and EE of Bi(Dig–PEG-PLGA)-S2 were more acceptable as compared with disulfide-linked redox-responsive systems designed for DOX delivery.^31,56^

### 3.6 Redox-Responsive Drug Releasing Behavior

**Figure 3:**
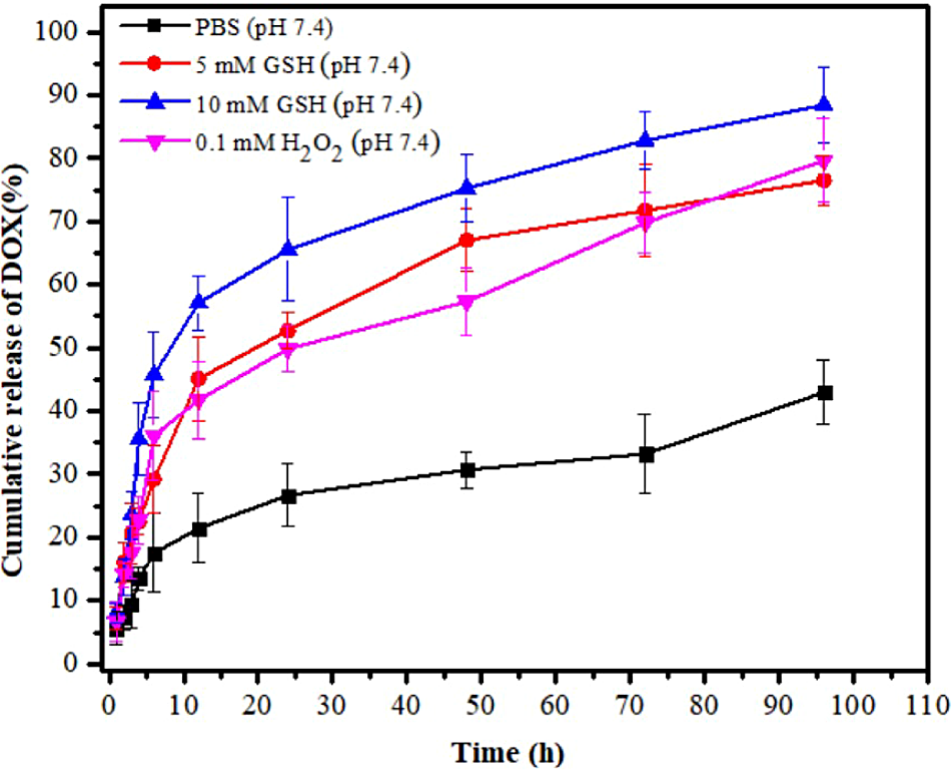
Drug release profile of DOX@Bi(Dig–PEG-PLGA)-S_2_ micelles under physiological and redox conditions.

In drug delivery applications, the release kinetics of payloads is crucial for achieving therapeutic concentration in the target cellular compartment and eliciting the intended bioactivity. Hence, stimuli-responsive drug delivery vehicles have become increasingly fabricated to ameliorate the anticancer activities of hydrophobic drugs such as DOX compared with conventional diffusion-triggered release systems. These vehicles offer spatiotemporal release kinetics in and around cancer cells. In this study, the release of DOX from the micelles was investigated under different conditions to mimic normal physiological conditions (PBS, *p*H 7.4) and cancer environments (5 mM GSH, 10 mM GSH, and 0.1 mM H_2_O_2_). The intracellular environment in cancer cells differs from that of normal cells (i.e., in cancer cells, the concentration of GSH and H_2_O_2_ exceeded by more than 1000 folds as compared to normal cells). This difference could contribute to micellar swelling or disassembly and controlled release of the cargo within cancer cells. In this regard, the high redox potential in cancer cells could result in cleavage and/or oxidation of disulfide bonds (S–S bond) in DOX@Bi(Dig–PEG-PLGA)-S_2_ micelles, which led to destabilization of micelles and resulted in site-specific release of DOX.^57−59^ As depicted in Figure 3, the release of DOX in reducing (5 mM GSH and 10 mM GSH) and oxidizing environments (0.1 mM H_2_O_2_), representing the cancer environment occurred much more abruptly than the release profile of DOX in PBS (pH 7.4), a normal physiological environment. The cumulative release of DOX (65.51 ± 8.21%) at higher concentrations of GSH (10 mM) within the first 24 h was significantly higher than the cumulative release of DOX (22.68 ± 5.01%) in the same time period in PBS. At 72 h, approximately 71.74 ± 7.22% and 82.78 ± 4.57% of DOX were released in GSH (5 mM) and GSH (10 mM) releasing media, respectively. After 96 h, nearly all DOX (89.41 ± 6.09%) was evacuated from DOX@Bi(Dig–PEG-PLGA)-S_2_ micelles into the surrounding 10 mM GSH-containing releasing media. Similarly, the cumulative release of DOX in the presence of 0.1 mM H_2_O_2_ at 96 h was about 79.64 ± 6.68%. In this circumstance, the amount of DOX released in the cancer redox environment was therapeutically sufficient for inducing necrosis of cancer cells even in the first 24 h. Interestingly, the cumulative release of DOX in PBS release medium was only 37.95 ± 5.08% within 96 h, suggesting minimal drug leakage under normal physiological conditions, thus minimizing undesired side effects on normal cells. The results are in line with the redox-responsive behavior of polymeric nanocarriers reported elsewhere.^31,60^ The redox-responsive behavior of the blank copolymer was further evaluated by estimating the molecular weight or elution time of the copolymer from APC after treatment with GSH. A concentration of 4 mg/mL of Bi(HOOC–PEG-PLGA)-S_2_ was treated with 5 mM GSH overnight, and then its molecular weight was examined using APC. As depicted in Figure S5, the APC tracing graph showed a bimodal distribution with a halved molecular weight, indicating the cleavage of disulfide bonds with GSH. On the other hand, the control group (Bi(HOOC–PEG-PLGA)-S_2_ in PBS) showed a unimodal peak with no change in its molecular weight.

### 3.7 Cellular Internalization of DOX-Loaded Micelles

**Figure 4:**
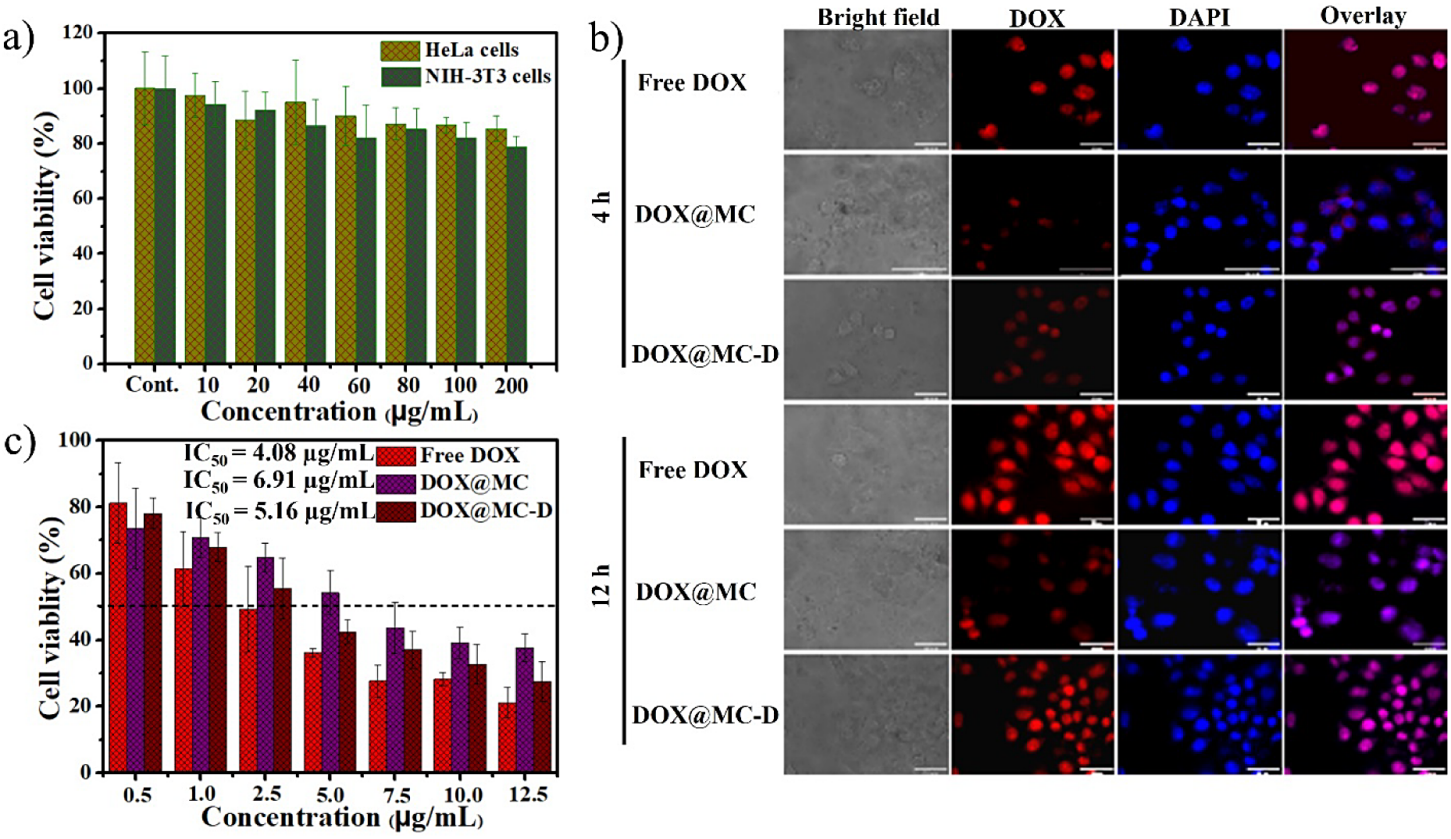
*In vitro* cellular experiments. a) Cytotoxicity of Bi(Dig–PEG-PLGA)-S_2_ copolymer against HeLa and NIH-3T3 cells; b) cellular uptake of free DOX, DOX@Bi(HOOC–PEG-PLGA)-S_2_, and DOX@Bi(Dig–PEG-PLGA)-S_2_ on HeLa cells (scale bar: 50 μm); c) anticancer activities of free DOX, DOX@Bi(HOOC–PEG-PLGA)-S_2_, and DOX@Bi(Dig–PEG-PLGA)-S_2_ on HeLa cells (**NB**: DOX@MC: DOX@Bi(HOOC–PEG-PLGA)-S_2_ micelle; DOX@MC-D: DOX@Bi(Dig–PEG-PLGA)-S_2_ micelle).

The cellular internalization of DOX@Bi(Dig–PEG-PLGA)-S_2_ micelles was determined qualitatively by tracing the red fluorescent intensity of DOX in the cytosol and nuclei as compared to that of DAPI (a blue fluorescent dye used to stain nuclei) using a fluorescent microscope. The overlapping of red fluorescence and blue fluorescence in the microscopic cell imaging referred to the internalization of the DOX@Bi(Dig–PEG-PLGA)-S_2_ micelles and subsequent delocalization of DOX to the nuclei.^61^ As shown in Figure 4b, the intensity of red fluorescence of cells treated with free DOX, DOX@Bi(HOOC–PEG-PLGA)-S_2_, and DOX@Bi(Dig–PEG-PLGA)-S_2_, with an equivalent DOX dose of 5.5 μg/mL, was time-dependent. Cells treated for 12 h showed relatively more intensified red fluorescence than the corresponding cells treated for 4 h. As the incubation time increased, the cellular internalization and cytosolic redox pool-triggered release of DOX from the micelle and its subsequent translocation to the nuclei could increase. Interestingly, the intensity of red fluorescence of cells treated with DOX@Bi(Dig–PEG-PLGA)-S_2_ was higher than that of DOX@Bi(HOOC–PEG-PLGA)-S_2_-treated cells, suggesting that the cellular trafficking of DOX@Bi(Dig–PEG-PLGA)-S_2_ was enhanced by the targeting ability of the digoxin moiety. The plasma membrane of HeLa cells has an overexpressed Na^+^/K^+^ ATPase that might enhance the cellular internalization of digoxin-modified micelles. Unlike DOX@Bi(HOOC–PEG-PLGA)-S_2_-treated cells, the red florescence intensity of DOX in HeLa cells incubated with DOX@Bi(Dig–PEG-PLGA)-S_2_ was superimposed with the blue fluorescence intensity of DAPI, asserting that the release and translocation of DOX from DOX@Bi(Dig–PEG-PLGA)-S_2_ micelles to the nuclei was higher than DOX@Bi(HOOC–PEG-PLGA)-S_2_ micelles. This important phenomenon reiterated the selective targeting ability of digoxin-modified micelles and the controlled release of DOX in the redox environment of cancer cells. The relatively smaller size of DOX@Bi(Dig–PEG-PLGA)-S_2_ micelles than DOX@Bi(HOOC–PEG-PLGA)-S_2_ micelles might also play a part in the enhanced crossing of the cell membrane. However, the red fluorescence intensity of cells treated with DOX@Bi(Dig–PEG-PLGA)-S_2_ was slightly weaker than that of cells treated with free DOX. The rapid and simple diffusion phenomenon of free DOX could possibly contribute to the abundant intracellular DOX levels, as manifested by the intensified red fluorescence in the nuclei of HeLa cells (treated with free DOX). Overall, the presence of digoxin as a targeting moiety and disulfide linkage as a redox- responsive spot had a profound effect on the cellular trafficking and accumulation of DOX@Bi(Dig–PEG-PLGA)-S_2_ micelles in cancer cells.

### 3.8 Cytotoxicity of Blank and DOX-Loaded Micelles

The biocompatibility of copolymers used as nanocarriers is an important concern in DDSs.^62^ Therefore, the safety profile of the block copolymer, Bi(Dig–PEG-PLGA)-S_2_, was assessed *in vitro* using normal (NIH-3T3) and cancer (HeLa) cell lines prior to anticancer activity testing. As shown in Figure 4a, the MTT assay revealed that the viability of both cell lines (NIH-3T3 and HeLa cells) treated with Bi(Dig–PEG-PLGA)-S_2_ for 24 h was remarkably high (≥80%), even at a maximum concentration of 200 μg/mL. The result reiterated that the Bi(Dig–PEG-PLGA)-S_2_ copolymer is devoid of notable cytotoxic effects against normal and cancer cells, making it safe and convenient for drug delivery applications. Inspired by the aforementioned scenario, the *in vitro* anticancer activity of DOX@Bi(HOOC–PEG-PLGA)-S_2_ and DOX@Bi(Dig–PEG-PLGA)-S_2_ was studied against HeLa cells. Following a 24 h treatment of HeLa cells with free DOX (positive control), the DOX@Bi(HOOC–PEG-PLGA)-S_2_ and DOX@Bi(Dig–PEG-PLGA)-S_2_ in a dose-dependent manner (equivalent DOX concentration ranging from 0.5 to 12.5 μg/mL), MTT assay was performed. As depicted in Figure 4c, the viability of cells treated with DOX@Bi(Dig–PEG-PLGA)-S_2_ was significantly lower (∼27%) than cells treated with DOX@Bi(HOOC–PEG-PLGA)-S_2_ (∼38%) at a maximum dose of DOX (12.5 μg/mL) (*p* < 0.05). However, the viability of cells treated with DOX@Bi(Dig–PEG-PLGA)-S_2_ was relatively comparable with that of cells treated with free DOX at equivalent doses. This might be attributed to the digoxin-directed enhancement of cellular internalization in cells treated with DOX@Bi(Dig–PEG-PLGA)-S_2_ and the sensitization of HeLa cells toward DOX by digoxin.^63^ The result was in line with the cellular uptake of DOX@Bi(Dig–PEG-PLGA)-S_2_ (Figure 4b), which was comparatively higher than that of DOX@Bi(HOOC–PEG-PLGA)-S_2_ and obviously might trigger subsequent cellular cytotoxicity. The half maximal inhibitory concentration (IC_50_) of DOX@Bi(Dig–PEG-PLGA)-S_2_, DOX@Bi(HOOC–PEG-PLGA)-S_2_, and free DOX were 5.16 μg/mL, 6.91 μg/mL, and 4.08 μg/mL, respectively. The relatively higher IC_50_ values of DOX@Bi(HOOC–PEG-PLGA)-S_2_ and DOX@Bi(Dig–PEG-PLGA)-S_2_ than the free DOX might be due to difference in the cellular trafficking mechanisms (diffusion versus receptor-mediated endocytosis) and relatively slow release rate of DOX from the micelles.^26,64^

### 3.9 Apoptosis Assays of DOX-Loaded Micelles

**Figure 5:**
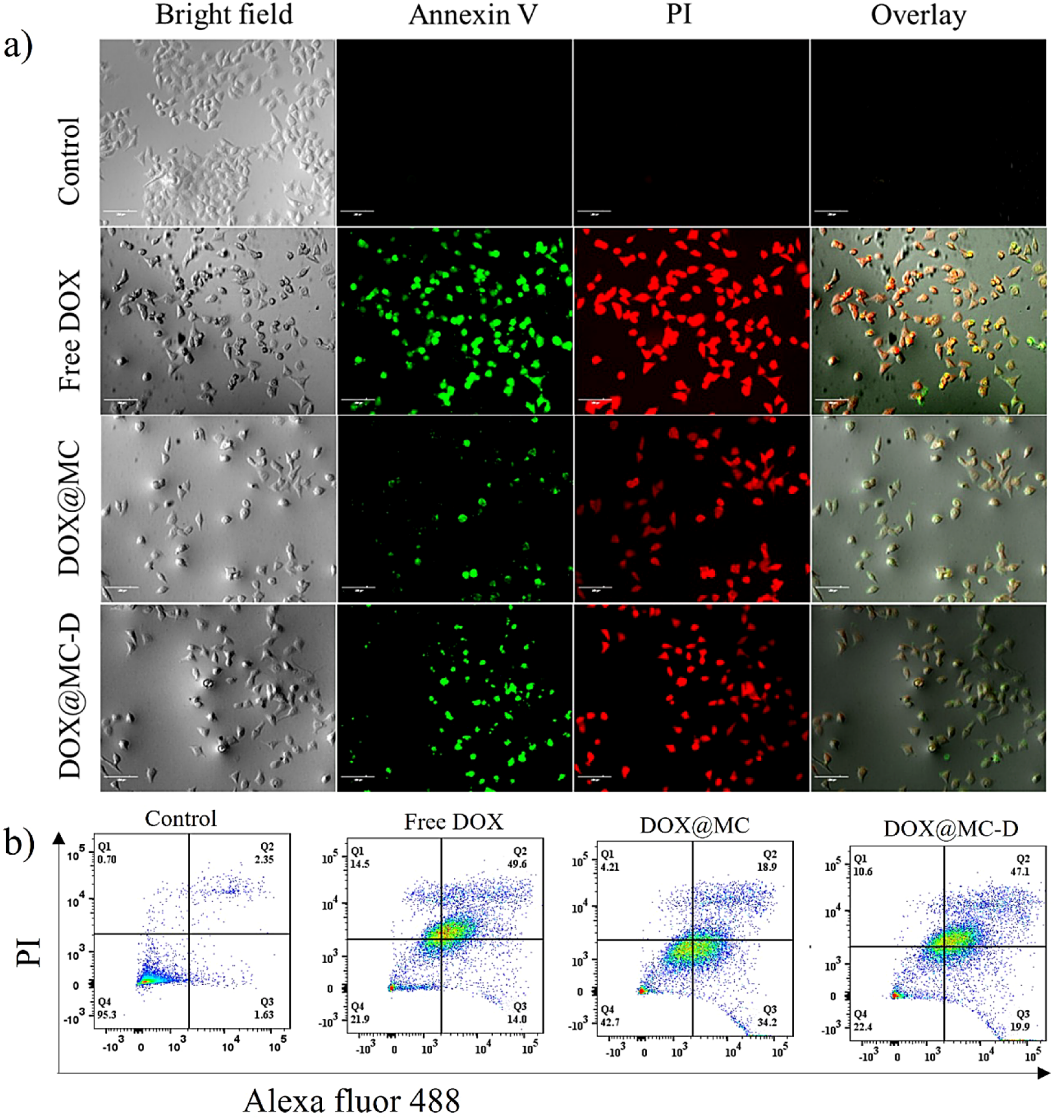
Apoptosis test. a) Fluorescence images of HeLa cells stained with annexin V/PI (scale bar: 50 μm) and b) quantitative estimation of apoptotic cells from FACS assay (NB: DOX@MC: DOX@Bi(HOOC–PEG-PLGA)-S_2_ micelle; DOX@MC-D: DOX@Bi(Dig–PEG-PLGA)-S_2_ micelle).

To complement the MTT assay, an *in vitro* apoptotic assay was implemented in our study. The apoptosis of cells induced by DOX@Bi(Dig–PEG-PLGA)-S_2_ in comparison to DOX@Bi(HOOC–PEG-PLGA)-S_2_ and free DOX against HeLa cells was studied through annexin V/PI double staining using confocal laser scanning microscopy (CLSM) imaging of apoptotic and necrotic cells after 12 h treatment with a corresponding DOX concentration of 5.5 μg/mL. As shown in Figure 5a, the intensity of green fluorescence was remarkably higher on cells treated with DOX@Bi(Dig–PEG-PLGA)-S_2_ than DOX@Bi(HOOC–PEG-PLGA)-S_2_, signifying that more cells undergo apoptosis by the synergistic effect of DOX and digoxin. Moreover, the presence of digoxin on the surface of the DOX@Bi(Dig–PEG-PLGA)-S_2_ micelles improved intracellular accumulation through digoxin-receptor-mediated endocytosis and concurrent release of DOX in the cytosol of HeLa cells, followed by a substantial cellular apoptosis.^65,66^

### 3.10 Flow Cytometry Analysis of DOX-Loaded Micelles

To further investigate the mechanisms involved in the therapeutic efficacy of DOX@Bi(Dig–PEG-PLGA)-S_2_ against HeLa cells, flow cytometry analysis was performed, including free DOX and DOX@Bi(HOOC–PEG-PLGA)-S_2_ for comparison. After 12 h of incubation with free DOX, DOX@Bi(HOOC–PEG-PLGA)-S_2_, and DOX@Bi(Dig–PEG-PLGA)-S_2_ at an equivalent DOX concentration of 5.5 μg/mL, HeLa cells were double stained with PI and annexin V-Alexa Flour 488, and the apoptotic and necrotic cells were quantified in terms of percentage. As depicted in Figure 5b, the percentage of HeLa cells undergoing early/late apoptosis was notably higher for cells treated with DOX@Bi(Dig–PEG-PLGA)-S_2_ compared to nontreated cells (negative control) or cells treated with DOX@Bi(HOOC–PEG-PLGA)-S_2_ under normal physiological conditions (37 °C and *p*H 7.4). The total percentage of early/late apoptotic cells in the DOX@Bi(Dig–PEG-PLGA)-S_2_-treated group was 67% (19.9% early apoptotic cells and 47.1% late apoptotic cells), which was higher than the total apoptotic cells of the DOX@Bi(HOOC–PEG-PLGA)-S_2_-treated group (34.2% early apoptotic cells and 18.9% late apoptotic cells). This suggested that the targeting moiety, digoxin, in DOX@Bi(Dig–PEG-PLGA)-S_2_ most likely increased cellular internalization of DOX@Bi(Dig–PEG-PLGA)-S_2_ and the subsequent cleavage and/or oxidation of S–S bonds in the micelles due to high levels of redox pool in the cytoplasm of cancer cells, resulting in swelling or disassembly of micelles and prompt release of DOX in the cells to induce apoptosis. Moreover, the anticancer and chemo-sensitizing effects of digoxin may have resulted in synergistic apoptotic effect on HeLa cells.^67^ These results align with the data obtained from CLSM imaging and the MTT assay (Figure 4b,c). On the other hand, unlike positive control cells (free DOX-treated cells) with a relatively higher percentage of early/late apoptotic cells (63.6%) and necrotic cells (14.5%), the total number of early/late apoptotic cells in the negative control cells (nontreated cells) was insignificant (3.98%). Overall, this study revealed that Bi(Dig–PEG-PLGA)-S_2_ micelles could serve as multifunctional nanocarriers that could potentially improve the efficacy and safety of chemotherapeutic drugs owing to the cancer cell recognition ability and synergistic chemotherapeutic effects for cancer treatment.

## 4 Conclusion

In conclusion, this study features the potential of redox-responsive Bi(Dig–PEG-PLGA)-S2 micelles as an effective drug delivery system for cancer treatment. These micelles, synthesized from an amphiphilic block copolymer, reveal selective accumulation in the tumor tissues via receptor-mediated endocytosis and respond to the unique redox environment of the tumor tissue. Moreover, the integration of digoxin improves the therapeutic efficiency of DOX, leading to rapid apoptosis of cancer cells. With desirable drug loading capacity and controlled release of DOX triggered by the cancer cell redox pool, Bi(Dig–PEG-PLGA)-S2 micelles significantly improve the cellular accumulation, temporal release, and subsequent cytotoxicity of DOX in cancer cells. Overall, our findings revealed that Bi(Dig–PEG-PLGA)-S2 micelles showed a promising drug delivery system for cancer therapy.

## Supporting Information Available

Scheme of stepwise synthesis of Bi(Dig–PEG-PLGA)-S_2_ copolymer; H1 NMR and APC characterization of HOOC–PEG-PLGA and Bi(HOOC–PEG-PLGA)-S2; the photoluminescence (Pl) spectra of pyrene in the micelles and determination of CMC for Bi (Dig–PEG-PLGA)-S2 copolymer; schematic illustration for DOX-loaded micelles preparation; UV–vis spectroscopy for estimation of standard calibration curve of free DOX and APC tracing to determine the redox responsiveness of Bi(HOOC–PEG-PLGA)-S2 (PDF)
