# Supplementary material for: Actively Targeting Redox-Responsive Multifunctional Micelles for Synergistic Chemotherapy of Cancer
Source: ACS Omega. 2024 Jul 29;9(32):34268–80. doi: 10.1021/acsomega.3c09817 (PMC11325410; doi:10.1021/acsomega.3c09817)
Supplement: Supplementary file 1 — ao3c09817_si_001.pdf [file ao3c09817_si_001.pdf]

Supplementary material

Actively targeting redox-responsive multi-functional micelles for synergistic chemotherapy of cancer

Haile Fentahun Darge [a,b,c], *Kefyalew Dagnew Addisu* [a,d], *Hsieh-Chih Tsai* [a,e,f,*], *Yihenew Simegniew Birhan* [a,g], *Endris Yibru Hanurry* [a,h], *Tefera Worku Mekonnen* [a], *Hailemichael Tegenu Gebrie* [a], *Vinothini Arunagiri* [a], *Darieo Thankachan* [a], *Tsung-Yun Wu* [a], *Juin-Yih Lai* [a,e,f], *Hao-Ming Chang* [i], *Chun-Chiang Huang* [j], *Szu-Yuan Wu* [k,l,m,n,o,p,q]*

[a] Graduate Institute of Applied Science and Technology, National Taiwan University of Science and Technology, Taipei 10607, Taiwan, ROC
[b] College of Medicine and Health Science, Bahir Dar University, Bahir Dar, P.O.Box 79, Ethiopia
[c] Centre for Ocular Research & Education (CORE), School of Optometry and Vision Science, University of Waterloo, 200 Columbia St W.,Waterloo, Canada
[d] Institute of Technology, Bahir Dar University, Bahir Dar, P.O.Box 79, Ethiopia
[e] Advanced Membrane Materials Center, National Taiwan University of Science and Technology, Taipei 10607, Taiwan, ROC
[f] R&D Center for Membrane Technology, Chung Yuan University, Chung-Li, Taiwan
[g] Department of Chemistry, College of Natural and Computational Sciences, Debre Markos University, P.O. Box 269, Debre Markos, Ethiopia.
[h] School of Medicine, Health Science College, Addis Ababa University, P.O.Box, 1176, Ethiopia
[i] Division of General Surgery, Tri-Service General Hospital, National Defense Medical Center, Taipei 114, Taiwan
[j] Taiwan Instrument Research Institute, National Applied Research Laboratories, Hsinchu, Taiwan
[k] Department of Food Nutrition and Health Biotechnology, College of Medical and Health Science, Asia University, Taichung, 413, Taiwan
[l] Big Data Center, Lo-Hsu Medical Foundation, Lotung Poh-Ai Hospital, Yilan, 256, Taiwan
[m] Division of Radiation Oncology, Department of Medicine, Lo-Hsu Medical Foundation, Lotung Poh-Ai Hospital, Yilan, 256, Taiwan
[n] Department of Healthcare Administration, College of Medical and Health Science, Asia University, Taichung, 413, Taiwan
[o] Cancer Center, Lo-Hsu Medical Foundation, Lotung Poh-Ai Hospital, Yilan, 256, Taiwan
[p] Graduate Institute of Business Administration, Fu Jen Catholic University, Taipei, 242, Taiwan
[q] Centers for Regional Anesthesia and Pain Medicine, Taipei Municipal Wan Fang Hospital, Taipei Medical University, Taipei, 110, Taiwan

* Correspondence: h.c.tsai@mail.ntust.edu.tw (H.C. Tsai), szuyuanwu5399@gmail.com (S.Y. Wu)

Table of content **Page**

Scheme S1: Stepwise synthesis of Bi(Dig-PEG-PLGA)-S_2 copolymer ……………………..…...3

Figure S1: Characterization of HOOC-PEG-PLGA and Bi(HOOC-PEG-PLGA)-S_2 ……..…4

Figure S2: Determination of CMC for Bi (Dig-PEG-PLGA)-S_2 copolymer …………..….....5

Figure S3: Schematic illustration of preparation of DOX-loaded micelles ………. ….. ….…5

Figure S4: Standard calibration curve estimation for free DOX. ……………………..…......6

Figure S4: Determination of redox responsiveness of Bi(HOOC-PEG-PLGA)-S_2 ………......7

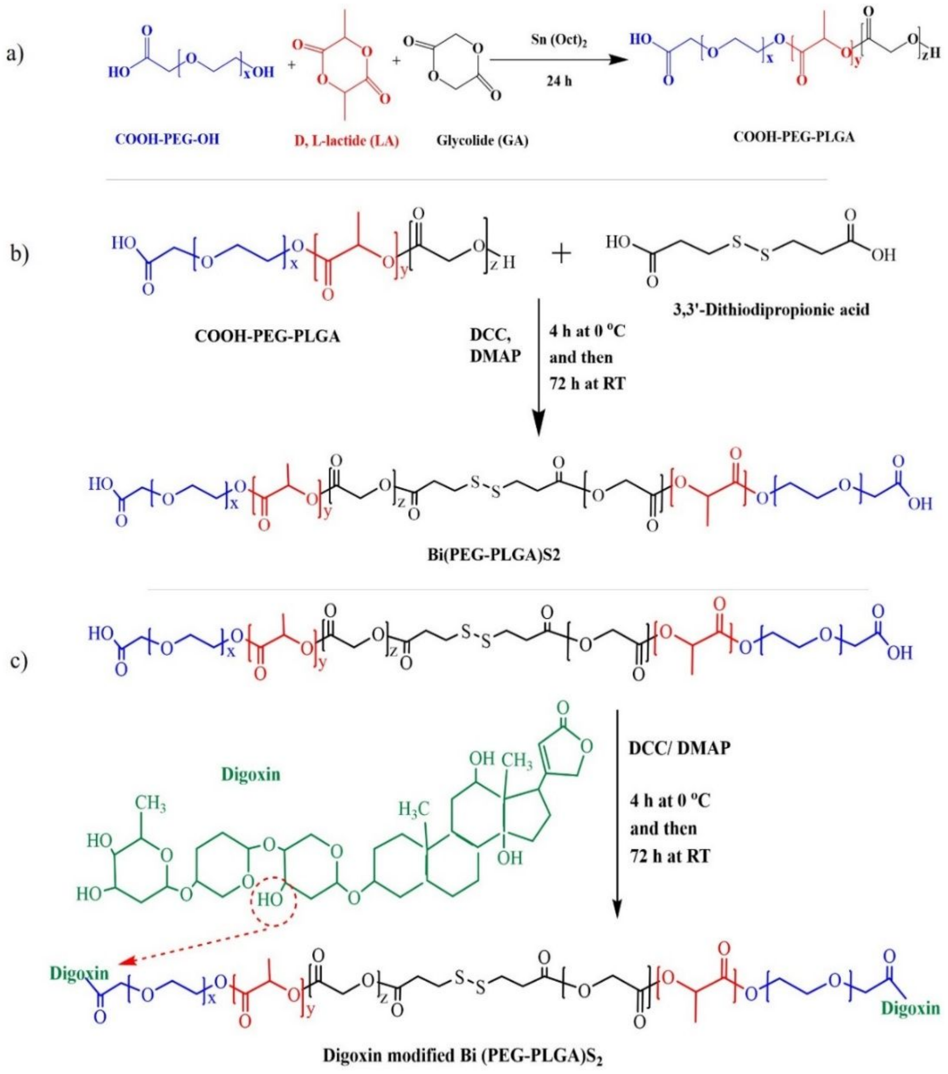

Scheme S1. Synthesis schemes of the copolymers. a) synthesis of HOOC-PEG-PLGA, b) Bi(HOOC-PEG-PLGA)-S_2, and c) Bi(Dig-PEG-PLGA)-S_2 copolymer.

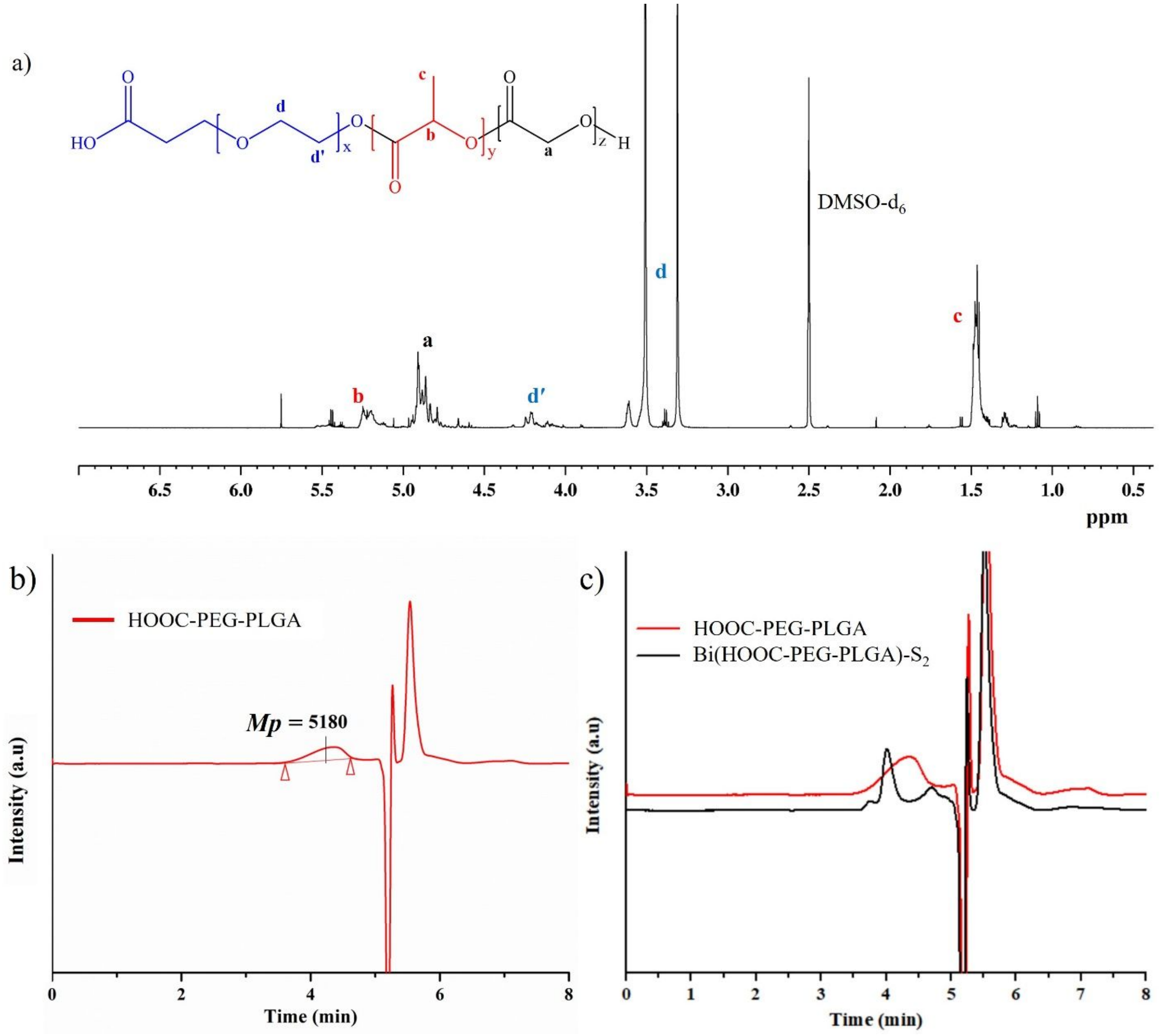

Figure S1. ^{1}H NMR of HOOC-PEG-PLGA (a) and APC tracing to measure the molecular weight of HOOC-PEG-PLGA and Bi(HOOC-PEG-PLGA)-S_2 (b & c)

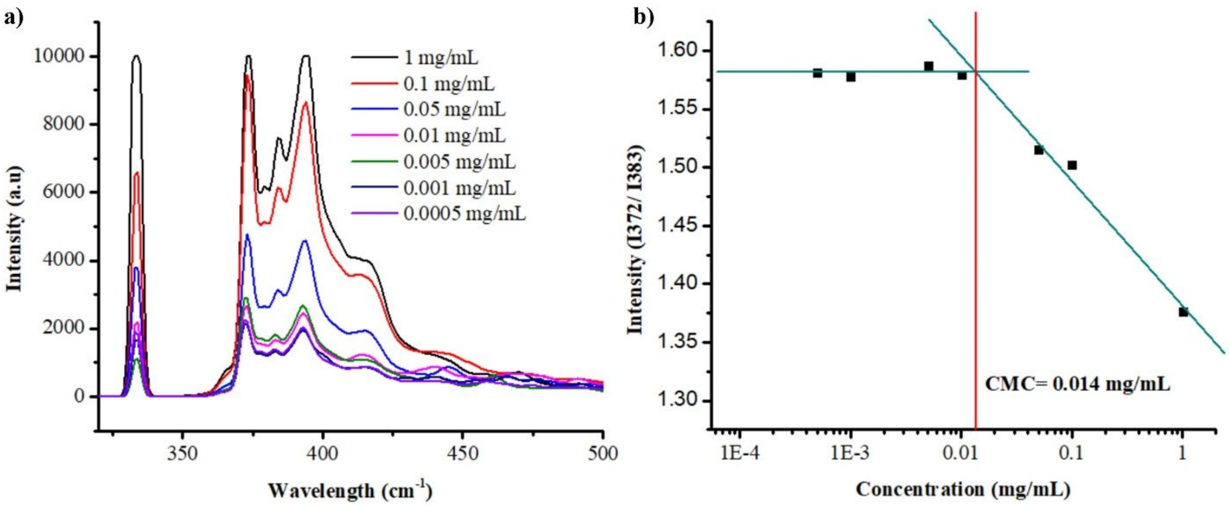

Figure S2. The photoluminescence (Pl) spectra of pyrene at different serial concentration of Bi (D-PEG-PLGA)-S_2 copolymer (a) and the critical micelle concentration (CMC) of Bi (Dig-PEG-PLGA)-S2 copolymer (b).

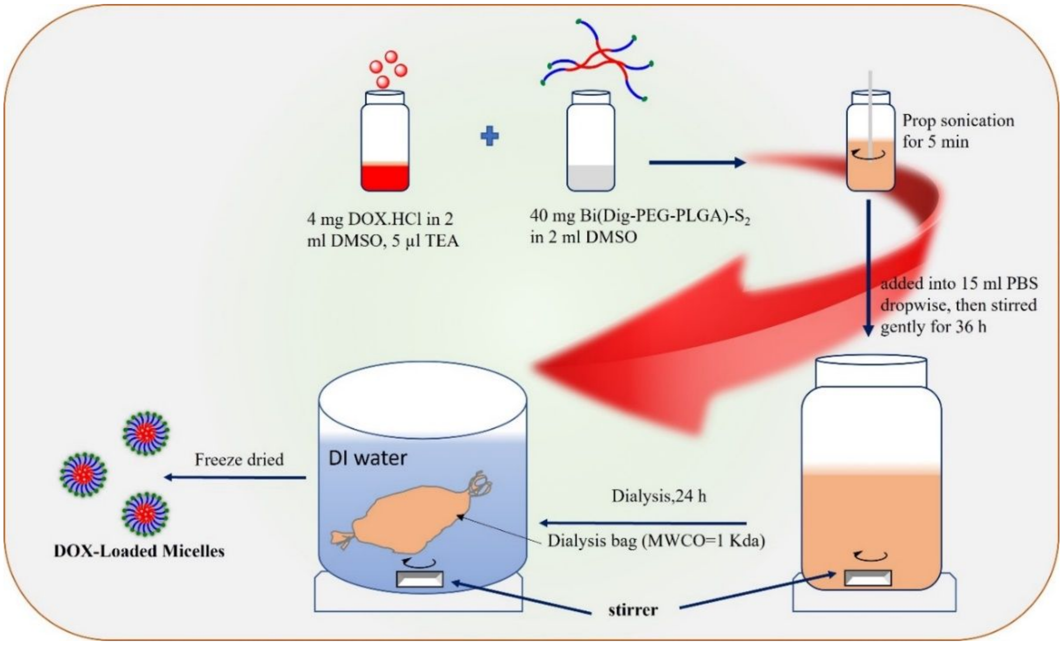

Figure S3. Schematic illustration of preparation of DOX-loaded micelles form the Bi(Dig-PEG-PLGA)-S_2 copolymer.

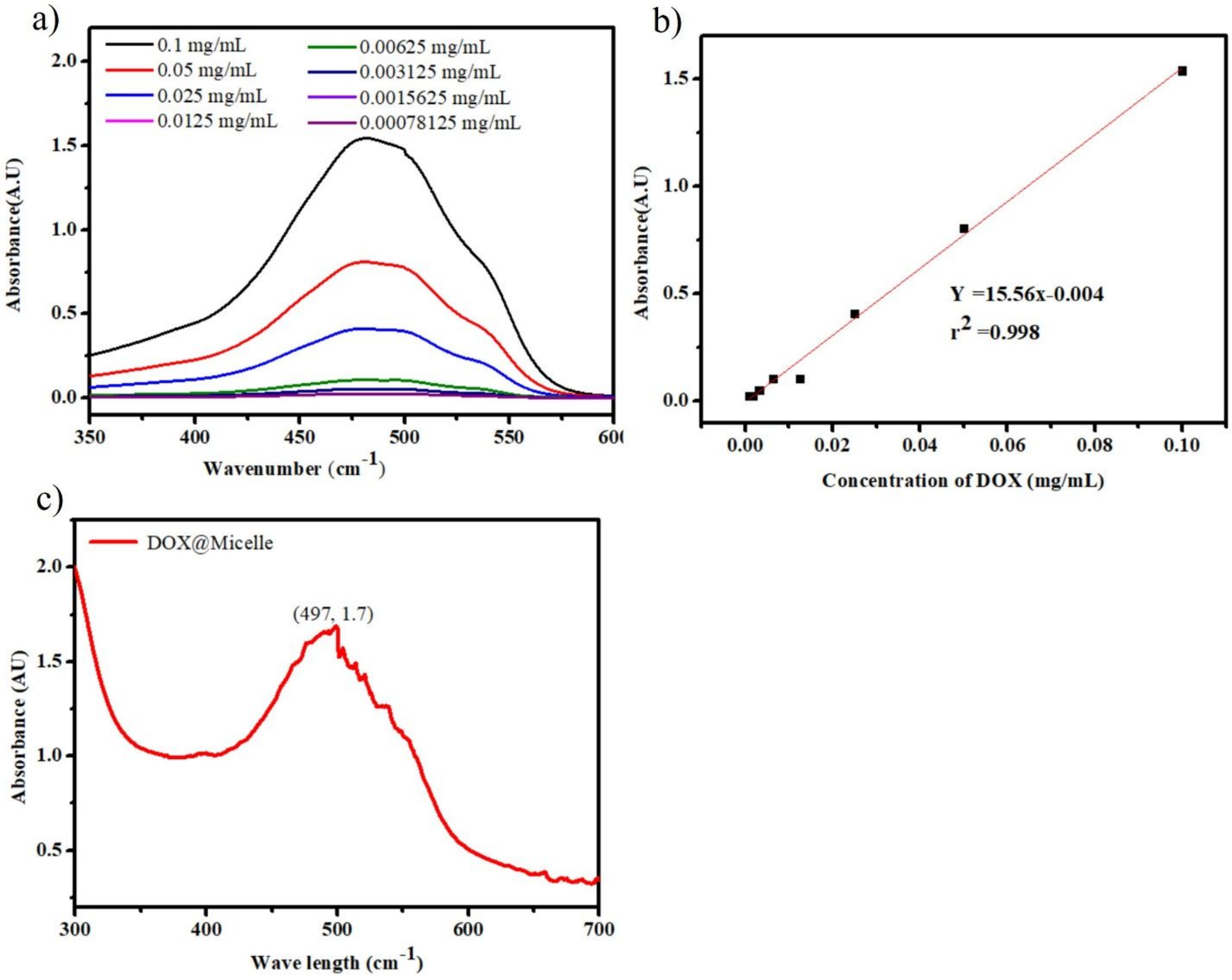

Figure S4. The UV-Vis absorbance of standard free DOX (a) Calibration curve for standard free DOX (b) and the UV-Vis absorbance of DOX loaded micelles (c).

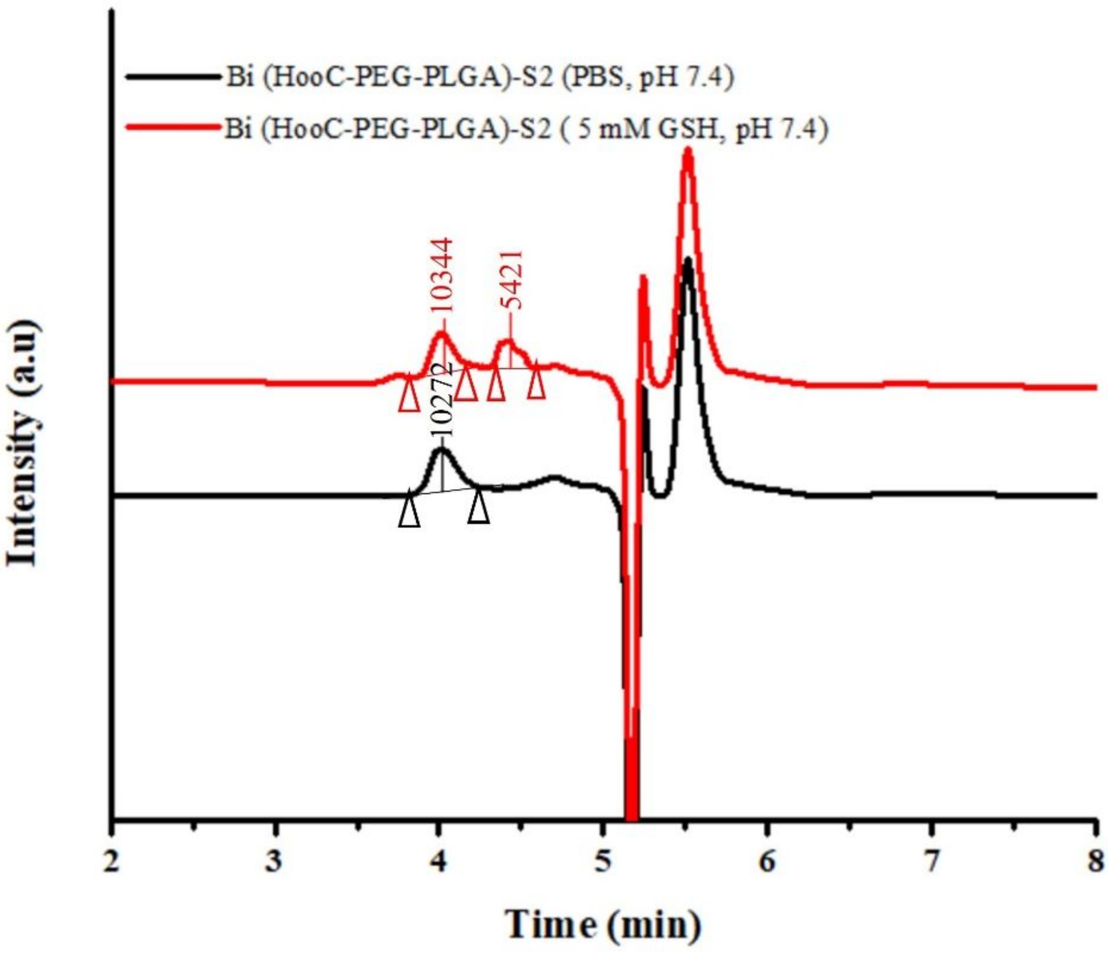

Figure S5. APC tracing determines the molecular weight of Bi(HOOC-PEG-PLGA)-S_2 after being treated with 5 mM GSH as reducing agent overnight. Bi(HOOC-PEG-PLGA)-S_2 in PBS was used as a control group.
